# Homologous human milk supplement for very low birth weight preterm infant feeding

**DOI:** 10.1016/j.rpped.2014.07.001

**Published:** 2015-03

**Authors:** Thayana Regina de Souza Grance, Paula de Oliveira Serafin, Débora Marchetti Chaves Thomaz, Durval Batista Palhares

**Affiliations:** Universidade Federal de Mato Grosso do Sul, Campo Grande, MS, Brazil

**Keywords:** Very low-birth weight infant, Food supplements, Human milk

## Abstract

**OBJECTIVE:**

To develop a homologous human milk supplement for very low-birth weight infant feeding, using an original and simplified methodology, to know the nutritional composition of human milk fortified with this supplement and to evaluate its suitability for feeding these infants.

**METHODS:**

For the production and analysis of human milk with the homologous additive, 25 human milk samples of 45mL underwent a lactose removal process, lyophilization and then were diluted in 50mL of human milk. Measurements of lactose, proteins, lipids, energy, sodium, potassium, calcium, phosphorus and osmolality were performed.

**RESULTS:**

The composition of the supplemented milk was: lactose 9.22±1.00g/dL; proteins 2.20±0.36g/dL; lipids 2.91±0.57g/dL; calories 71.93±8.69kcal/dL; osmolality 389.6±32.4mOsmol/kgH_2_O; sodium 2.04±0.45mEq/dL; potassium 1.42±0.15mEq/dL; calcium 43.44±2.98mg/dL; and phosphorus 23.69±1.24mg/dL.

**CONCLUSIONS:**

According to the nutritional contents analyzed, except for calcium and phosphorus, human milk with the proposed supplement can meet the nutritional needs of the very low-birth weight preterm infant.

## Introduction

Although it does not fully meet the nutritional needs of very low birth weight (VLBW) infants, human milk is recommended considering the digestive, metabolic and immunological immaturity of these children.^1^^,^ 2 The American Academy of Pediatrics, as well as the Brazilian Ministry of Health^3^^,^ 4 recognize that human milk is beneficial and has many advantages for preterm infant feeding. However, due to its physiological characteristics, human milk supplementation is recommended for these newborns.^5^^-^ 8

Considering its greater availability, cow's milk protein is the most commonly used human milk supplement. The concern with the short-term prognosis and the knowledge that nutrition in childhood is related to diseases of adulthood justify the study of supplement use that offers more adequate quality amino acids and fatty acids, such as those derived from human milk itself.^9^^-^ 14

These studies show that it is possible to offer higher concentrations of human milk nutrients to VLBW infants with good gastrointestinal and metabolic tolerance. Among such studies, we highlight those using viable methodologies to be applied in human milk banks, such as evaporation and freeze drying of skim or non-skim human milk after removal of part of the lactose.^9^^-^ 14 In these studies, human milk was concentrated in a rotary evaporator, a procedure that requires the milk to be on average 30 minutes at a temperature higher than room temperature, which in addition to requiring extensive manipulation of the milk, allows greater protein denaturation due to time and temperature range.

By analyzing the techniques used in these studies, it was intended to develop a homologous human milk supplement for VLBW infant feeding with an original, simplified methodology, which minimized the steps of handling and temperature change such as heating, freezing and thawing during its production. We also sought to assess the nutritional composition of human milk fortified with this supplement and evaluate its suitability for the feeding of these newborns. 

## Method

After approval by the Human Research Ethics Committee at Universidade Federal de Mato Grosso do Sul (UFMS) (n. 1975 CAAE 0035.0.049.000-11), the homologous supplement of human milk was prepared.

The human milk used in the production and dilution of the supplement and the one used for comparison with the supplemented milk was expressed manually at home or in the human milk bank at the University Hospital of UFMS, being donated by volunteer mothers whose children were born at full-term, with a lactation period between 0 and 12 months. Milk samples that were submitted to the selection criteria of the human milk bank and that showed an index ≤2 in the acidity titration analysis (Dornic acidity) were used for supplement production.^15^ Milk samples used in the study were not selected according to lactation period, beginning or end of feeding, or time of day when the sample was collected. Although all these characteristics influence the composition of human milk, it is known that human milk banks usually have no surplus milk supply, and therefore this study did not aim to work with selected milk samples.

The human milk samples underwent two phases of preparation: removal of lactose and lyophilization, as described below. First, 25 samples of human milk with 45mL were placed in conical plastic tubes, refrigerated at -22 °C for 24 hours. After this period, the samples were submitted to centrifugation at 3,500 rpm for 60 minutes in a NT 815 (NOVATECNICA^(r)^, SP, Brazil) centrifuge at a temperature of 0-2 °C to allow the formation of lactose precipitate. The initial volume of 45mL was defined as it is the standard volume of the conical plastic tubes used in centrifuges. The samples were then heated in a water bath at 40 °C for the minimum time required for thawing to facilitate the standardized removal of 40mL of supernatant. The supernatant was then transferred to a glass container, using a graded pipette. The lactose deposit was discarded. The remaining contents of the lactose removal process were frozen again at -22 °C for 24 hours and placed in the vacuum chamber of the benchtop lyophilizer L101 (LIOBRAS^(r)^, SP, Brazil) for 72 hours. The lyophilization process results in milk dehydration by sublimation. The weight of each lyophilized sample varied according to the initial fat content, as the volume of fat does not change in lyophilization. Each lyophilized sample was diluted in 50mL of human milk. By diluting the supplement in 50mL of human milk, the goal was to have approximately a two-fold higher content of nutrients in the supplemented milk, as the supplement originated from 45mL. 

These samples were analyzed for the contents of lactose, protein, lipids, osmolality, sodium, potassium, calcium and phosphorous. The macronutrient measurement was performed in Nexgen equipment (Bentley^(r)^, MN, USA), osmolality was evaluated in a VAPRO osmometer (WESCOR^(r)^, UT, USA), sodium and potassium measurement was performed in a B262 flame photometer (Micronal^(r)^, SP, Brazil), and calcium and phosphorus were measured in a spectrophotometer, with sample ash solution, according to the methodology described by Instituto Adolfo Lutz.^16^ For the comparison with the 25 supplemented milk samples, macronutrients were measured in 20 samples of human milk without supplement. The mineral contents were compared with those reported by Palhares et al in 1987.^17^ The energy value of the samples was calculated in calories by multiplying each gram of carbohydrate and protein by 4, and each gram of fat by 9. 

During the supplement production, there was a concern regarding the maintenance of physicochemical quality and the lowest degree of microbiological contamination of the milk, as it may change the physicochemical aspects of milk. For that purpose, the following measures were taken: the containers that stored the milk during the two phases of preparation, as well as the volumetric pipette, were autoclaved; milk fractionation for supplement preparation and dilution was carried out after disinfecting hands, using gloves, masks, aprons and caps; the rim of the flasks were sterilized using the Bunsen burner after being opened and before being closed; the water bath used in sample thawing was previously cleaned and the water replaced for each preparation; the lyophilizer vacuum capsule was cleaned before and after use; the lyophilized supplement was stored with the cap on, under freezing conditions, in the same container in which it was lyophilized, until it was diluted. In this study, the VLBW infants were not fed with the supplemented milk, and therefore the supplemented milk did not undergo microbiological analysis. However, the supplement was designed to be added to raw human milk, which should be pasteurized after being supplemented and submitted to routine microbiological analysis of the human milk bank before being offered to newborns. 

In experimental studies, compared groups should have a sample size that is sufficient to identify the difference between them, i.e., the sample size is related to the expected variation between the assessed groups, in addition to the confidence interval.^18^ Thus, the sample size in this study was defined by taking into account research in the area and the expected difference between the analyzed groups. To compare the nutritional content of supplemented human milk with human milk without supplement, the nonparametric Wilcoxon test was used in the GraphPad Software 2013, (GraphPad Software, Inc^(c)^, CA, USA). The significance level was set at 5%.

## Results

The mean, standard deviation, minimum and maximum values of lactose, proteins, lipids, energy, osmolality, sodium, potassium, calcium and phosphorus of human milk with homologous supplement are shown in Table 1. Table 2 shows the mean concentrations of the nutritional content in human milk with homologous supplement compared to human milk without supplement. 

**Table 1:** Values of lactose, proteins, lipids, energy, osmolality, sodium, potassium, calcium and phosphorus in the homologous supplemented human milk.

Nutrient	Mean	SD	Minimum	Maximum
Lactose (g/dL)	9.22	1.00	7.80	11.02
Protein (g/dL)	2.20	0.36	1.60	2.90
Lipids (g/dL)	2.91	0.57	1.81	4.01
Energy (kcal/dL)	71.93	8.69	54.88	89.45
Osmolality (mOsm/KgH_2_O)	389.6	32.4	335.00	438.00
Sodium (mEq/dL)	2.04	0.45	1.30	2.60
Potassium (mEq/dL)	1.42	0.15	1.22	1.66
Calcium (mEq/dL)	43.44	2.98	38.07	48.21
Phosphorus (mEq/dL)	23.69	1.24	21.72	25.51
SD, standard deviation.				

SD, standard deviation.

**Table 2:** Mean nutritional content of homologous supplemented human milk (HM+HS) and human milk from a milk bank (HM).

Nutrient	HM+HS	HM	Increase in folds	p
Lactose (g/dL)	9.22	6.78	1.36	<0.001
Protein (g/dL)	2.20	1.13	1.95	<0.001
Lipids (g/dL)	2.91	1.49	1.95	<0.001
Energy (g/dL)	71.93	45.06	1.60	<0.001
Osmolality (mOsm/KgH_2_O)	389.6	267.70	1.45	<0.001
Sodium (mEq/dL)	2.04	0.93^17^	2.19	<0.001
Potassium (mEq/dL)	1.42	1.18^17^	1.20	<0.011
Calcium (mEq/dL)	43.44	24.71^17^	1.76	<0.001
Phosphorus (mEq/dL)	23.69	10.75^17^	2.20	<0.001

## Discussion

Human milk supplementation emerged with the aim of adapting it to the nutritional needs of the very low (VLBW) and extremely low birth weight (ELBW) preterm infants. The use of commercial fortifiers and lactoengeneering techniques of human milk, among them the adequate structuring of human milk banks, the breakdown of fat globules of human milk by ultrasound and the concentration of human milk through evaporation of milk stored in these banks, renewed the enthusiasm for the use of human milk to feed these children.^19^

The technique described in this study was designed to minimize the manipulation steps, changes in temperature and the physical state of milk to attain better nutrient conservation. The handling of milk is only performed in the steps of storing it in conical plastic tubes and removal of the supernatant. As for the temperature changes, before the supplement production process, it is frozen and thawed only once during the process (at the supernatant removal after lactose precipitation), when it is again frozen, freeze-dried at a temperature of -40 °C and maintained under freezing to be reconstituted in human milk. That is different from the techniques described in similar studies.^11^^-^ 14

Nutrient concentration in human milk and the use of supplements lead to increased osmolality. Therefore, part of the lactose was removed in the precipitation process. The content of lactose, which is a disaccharide, increases the solution's osmolality.^20,21^ On the other hand, lactose enhances calcium absorption and promotes fermentation, which reduces intestinal constipation.^22^^,^ 23 The mean content of lactose in supplemented human milk was 9.2g/dL, in accordance with the recommendation of 3 to 12g/dL. With the onset of minimal enteral feeding, the activity of lactase increases rapidly. Despite the increase in lactose content, osmolality was found to be 389.6mOsm/KgH_2_O. The osmolality of food for premature infants should be less than 450mOsm/kgH_2_O.^24^ Santos,^12^ in 1997, fed VLBW infants human milk with added homologous supplement whose lactose content was similar and reported good gastrointestinal tolerance. Thus, the lactose content found in this study seems to be adequate for feeding VLBW infants.

The amounts of proteins found in human milk with supplement meet the AAP recommendation,^25^ in 1985, of 2.9-3.3g/100kcal, and of the Canadian Society of Pediatrics,^26^ which recommended 2.7-3.5g/kg/day in 1995. With a supply of supplemented milk of 150-200mL/kg a day, the intake of 3.3-4.4g/kg of protein a day is attained, coincident with the interval of 3.5-4.0g/kg/day recommended by the European Society for Pediatric Gastroenterology, Hepatology, and Nutrition (ESPGHAN) in 2010.^27^

The sodium content found in supplemented milk also meets the recommendations of the AAP for infant formulas, which are 2.5-3.5mEq/kg/day, when 120kcal/kg/day are offered,^25^ and of ESPGHAN^26^ (2.7-4.6mEq/100kcal). If the child's gastric capacity allows it, the intake of a volume of milk that supplies 4mEq/kg/day of sodium is recommended for the prevention of hyponatremia.^25^ As for potassium, supplemented human milk in this study had 1.42mEq/dL of potassium. The estimated requirement of potassium can be supplied by breastfeeding, which contains 1.25-1.60mEq/dL.^28^

The presence of calcium in human milk differs from that in infant formulas, not only in quantity, but also regarding the constituent chemical species due to differences in the protein fraction of human and cow's milk. In formulas, calcium is mainly associated with casein. In human milk, a high proportion of calcium constitutes part of the lipid fraction. In the aqueous fraction, most of the calcium is associated with whey proteins and low-molecular weight compounds. In human milk there is little calcium bound to casein. These differences in the chemical structure of the constituent species of the calcium content of milk explain the high bioavailability of calcium in human milk.^28^ Despite the significant increase in the levels of calcium and phosphorus in supplemented human milk compared to human milk without supplement, these levels do not meet the recommendations for very low birth weight infants, which, according to ESPGHAN, are 20-140mg/kg/day of calcium and 60-90mg/kg/day of phosphorus,^27^ and according to AAP, are 200 to 250mg/kg/day of calcium and 110 to 125mg/kg/day of phosphorus.^25^ Still, this significant increase of approximately twice than that of human milk without supplements is advantageous due to the increased bioavailability of calcium and phosphorus in human milk when compared to commercial supplements. Additionally, the acquisition of calcium and phosphorus supplements alone is cheaper than purchasing multinutrient supplements. 

As the incorporation of fat in the fetus occurs in the last trimester, the VLBW infants are vulnerable to the lack of lipids. The fat in VLBW infants' diet must meet the needs of fatty acids and prevent lack of calories.^29^ The amount of fat in the human milk with homologous supplement in g/100kcal was 4g, close to the recommendations of the AAP, 4.5-6.0g/100kcal^25^ and of ESPGHAN,^27^ 4.4-6.0g/100kcal. The contents of fat in human milk homologous supplement and of the human milk without supplement shown in the results are lower than the mean fat content in human milk described in the literature. This occurred because the milks used were not selected according to their caloric value, due to the difficulty of having surplus milk in the milk bank for research. Nevertheless, the supplement made with these "lean" milks was able to significantly improve the nutritional content of human milk from the bank for VLBW infants.

The milk produced by the mother of a preterm newborn has a different composition from mature milk, regarding protein and energy content and immunological constituents in the first weeks of production. These modifications make breast milk adapted to the needs of the premature infant. In the first four weeks, preterm human milk contains a higher concentration of nitrogen, protein with immune function, total lipids, medium-chain fatty acids, vitamins, calcium, sodium and energy than milk produced by mothers of full-term newborns. The greater the degree of prematurity, the higher the lipid and protein content.^1^^,^ 30 With the exception of calcium and phosphorus, the nutrient levels evaluated in supplemented milk meet the recommendations for nutrition of VLBW infants and their composition is similar to preterm human milk, thus suitable for feeding these children. The result obtained in this study is similar to that of human milk with added supplements produced by Thomaz et al,^14^ and has a higher protein content than those produced by Santos^12^ and Valentini.^13^

The main limitations regarding the study methodology are its reproducibility, as the values of nutritional composition vary according to the human milk used; using different pools of human milk in the supplement production, dilution and comparison of their nutritional compositions, and the comparison of the mineral content with data from another study. Such limitations are justified below.

When reproducing the methodology used in this study, the results of the nutritional composition of human milk with or without supplement can vary according to the nutritional composition of the human milk used, which undergoes variations during the day, from one breast to another, from the beginning to the end of the feeding, and during the period of lactation. However, the main objective of this study is to show that the technique for supplement production is capable of making the milk from the milk bank more suitable for feeding VLBW infants without establishing selection and exclusion criteria for the milk, in addition to those that guarantee sanitary safety. This is due to the fact that, in human milk banks, there is usually no surplus breast milk, and thus a technique that establishes selection criteria for the available milk used in the production of the supplement would not be feasible. Because of this variation in availability of milk from the milk bank, in this study the milk used in the production of the supplement, in the dilution and comparison of their nutritional compositions did not originate from the same pool, but represented mean values of different samples. 

A comparison of the mineral content of human milk with homologous supplement with human milk without supplement was carried out with data published in another study, because the nutritional composition of human milk with no supplements is well described in the publications. 

Regarding the feasibility of the supplement production, the only initial cost to equip the milk bank and produce the homologous supplement is approximately U$19,000.00 (nineteen thousand American Dollars) to acquire the refrigerated centrifuge and the benchtop freeze-dryer. Considering that the market price of the commercial supplement box with 70 vials is R$115.00, and one vial is enough to supplement 20mL of human milk, the cost to feed a child weighing 1.000g with a daily volume of 150mL/kg for 30 days will be approximately R$370.00. To feed 10 children a day with this volume for 1 year, the cost will be R$44,400.00. Therefore, a neonatal unit that treats 10 children per month, using the proposed volume, will have spent in one year roughly the same amount needed to equip the human milk bank to produce the same homologous supplement.

The homologous supplement has advantages over commercial ones, as it provides protein of high biological value and nutrients of human milk. The technique can be implemented in human milk banks and has financial viability in developing countries, as it requires only an initial investment for the purchase of equipment and it reduces costs with commercial supplements.
