# Supplementary material for: Homologous human milk supplement for very low birth weight preterm infant feeding
Source: Rev Paul Pediatr. 2015 Mar;33(1):28–33. doi: 10.1016/j.rpped.2014.07.001 (PMC4436953; doi:10.1016/j.rpped.2014.07.001)
Supplement: Translation

# Translation: Aditivo homólogo para a alimentação do recém-nascido pré-termo de muito baixo peso

**Authors:** Thayana Regina de Souza Grance, Paula de Oliveira Serafin, Débora Marchetti Chaves Thomaz, Durval Batista Palhares

**Affiliations:** Universidade Federal de Mato Grosso do Sul (UFMS), Campo Grande, MS, Brasil

**Keywords:** Recém-nascido de muito baixo peso, Aditivos alimentares, Leite humano

## Abstract

**OBJETIVO:**

Elaborar com metodologia original e simplificada um aditivo homólogo do leite humano para a alimentação do recém-nascido de muito baixo peso, conhecer a composição nutricional do leite humano fortificado com esse aditivo e avaliar sua adequação para a alimentação desses recém-nascidos.

**MÉTODOS:**

Para a produção e análise do leite humano com o aditivo homólogo, 25 amostras de 45 mL de leite humano passaram por processos de retirada de lactose, liofilização e foram diluídas em 50 mL de leite humano. Foram feitas dosagens de lactose, proteínas, lipídios, energia, sódio, potássio, cálcio, fósforo e osmolalidade.

**RESULTADOS:**

A composição do leite aditivado foi lactose 9,22 ± 1 g/dL; proteínas 2,20 ± 0,36 g/dL; lípides 2,91 ± 0,57 g/dL; calorias 71,93 ± 8,69 kcal/dL; osmolalidade 389,6 ± 32,4mOsmol/kgH_2_O; sódio 2,04 ± 0,45mEq/dL; potássio 1,42 ± 0,15mEq/dL; cálcio 43,44 ± 2,98 mg/dL; e fósforo 23,69 ± 1,24 mg/dL.

**CONCLUSÕES:**

De acordo com os teores nutricionais analisados, com exceção do cálcio e do fósforo, o leite humano com o aditivo proposto pode atender às necessidades nutricionais do recém-nascido pré-termo de muito baixo peso.

## Introdução

Apesar de não atender plenamente àss necessidades nutricionais do recém-nascido de muito baixo peso (RNMBP), o leite humano é recomendado em vista da imaturidade digestiva, metabólica e imunológica que essas crianças apresentam.^1^ ^and^ 2 A Academia Americana de Pediatria, bem como o Ministério da Saúde brasileiro,^3^ ^and^ 4 reconhece que o leite humano é benéfico e apresenta inúmeras vantagens na alimentação de pré-termos. No entanto, por suas características fisiológicas, recomenda-se a suplementação do leite humano para esses recém-nascidos.^5^^,^ 6 ^,^ 7 ^and^ 8

Considerando sua maior disponibilidade, a proteína do leite de vaca é a mais frequentemente usada nos aditivos de leite humano. A preocupação com o prognóstico em curto prazo e o conhecimento de que a nutrição na infância se relaciona com doenças da vida adulta fizeram com que o uso de aditivos que ofereçam qualidade de aminoácidos e ácidos graxos mais adequados, como os derivados do próprio leite humano, esteja sendo estudado.^9^^,^ 10 ^,^ 11 ^,^ 12 ^,^ 13 ^and^ 14

Esses trabalhos mostram que há possibilidades de oferecer concentrações maiores de nutrientes do leite humano ao RNMBP com boa tolerância gastrointestinal e metabólica. Dentre tais estudos, destacam-se aqueles que usaram metodologias viáveis para serem aplicadas em bancos de leite humano, como evaporação e liofilização do leite humano desnatado ou não, após retirada de parte da lactose.^9^^,^ 10 ^,^ 11 ^,^ 12 ^,^ 13 ^and^ 14 Nesses trabalhos o leite humano foi concentrado em um rotaevaporador, procedimento que exige que o leite fique em média 30 minutos a uma temperatura maior do que a ambiente, o que, além de exigir grande manipulação do leite, possibilita maior desnaturação de proteínas pelo tempo e pela faixa de temperatura.

Na análise das técnicas usadas nestes estudos, pretendeu-se: elaborar um aditivo homólogo do leite humano para a alimentação do RNMBP com metodologia original, simplificada e que minimizasse etapas de manipulação e mudanças de temperatura como aquecimento, congelamento e descongelamento durante sua produção. Além disso, procurou-se conhecer a composição nutricional do leite humano fortificado com esse aditivo e avaliar sua adequação para a alimentação desses recém-nascidos.

## Método

Após a aprovação do Comitê de Ética em Pesquisas com seres humanos da Universidade Federal de Mato Grosso do Sul (UFMS) (n° 1.975 CAAE 0035.0.049.000-11), foi preparado o aditivo homólogo de leite humano.

O leite humano usado na produção e diluição do aditivo e aquele empregado na comparação com o leite aditivado foi ordenhado por meio de expressão manual em domicílio ou no banco de leite humano do Hospital Universitário da UFMS e doado por mães voluntárias, cujos filhos nasceram a termo, com período de lactação de até 12 meses. Para a produção do aditivo foram usados leites que passaram pelo critério de seleção do banco de leite humano^15^ e que obtiveram, na análise de titulação de acidez (acidez Dornic), índice ≤2. As amostras de leite usadas não foram selecionadas segundo tempo de lactação, início ou fim da mamada ou hora do dia em que a amostra foi coletada. Apesar de essas características influenciarem a composição do leite humano, sabe-se que os bancos de leite humano usualmente não têm estoque excedente de leite e, por isso, este trabalho não teve o objetivo de trabalhar com leites selecionados.

As amostras de leite humano passaram por duas fases de preparo: retirada de lactose e liofilização, descritas a seguir. Em primeiro lugar, foram acondicionadas 25 amostras de 45 mL de leite humano em tubos plásticos cônicos refrigerados a -22 °C por 24 horas. Após esse período, as amostras foram submetidas à centrifugação a 3.500 rpm durante 60 minutos em centrífuga NT 815 (Novatecnica^(r)^, SP, Brasil) na temperatura de 0-2 °C para formação de precipitado de lactose. O volume inicial de 45 mL foi definido porque é o volume padrão dos tubos plásticos cônicos usados na centrifuga. As amostras foram então aquecidas em banho-maria a 40 °C por tempo mínimo necessário ao descongelamento para facilitar a retirada padronizada de 40 mL do sobrenadante. O sobrenadante foi transferido para um recipiente de vidro, com o uso de uma pipeta graduada. O depósito de lactose formado foi desprezado. O conteúdo restante do processo de retirada da lactose foi congelado novamente a -22 °C por 24 horas e colocado na câmara de vácuo do liofilizador de bancada L101 (Liobras^(r)^, SP, Brasil) por 72 horas. O processo de liofilização promove a desidratação do leite por sublimação. O peso de cada amostra liofilizada varia de acordo com o teor inicial de gordura, uma vez que o volume dessa não se altera na liofilização. Cada amostra liofilizada foi diluída em 50 mL de leite humano. Ao diluir o aditivo em 50 mL de leite humano, o objetivo foi ter aproximadamente o dobro de nutrientes no leite aditivado, já que o aditivo originou-se de 45 mL.

Essas amostras foram analisadas quanto ao teor de lactose, proteínas, lipídios, osmolalidade, sódio, potássio, cálcio e fósforo. A dosagem de macronutrientes foi feita no aparelho Nexgen (Bentley^(r)^, MN, EUA), a osmolalidade foi avaliada no aparelho osmômetro Vapro (Wescor^(r)^, UT, EUA), a dosagem de sódio e potássio foi feita no fotômetro de chama B262 (Micronal^(r)^, SP, Brasil) e a dosagem de cálcio e fósforo, no espectrofotômetro com a solução de cinzas das amostras, conforme metodologia descrita do Instituto Adolfo Lutz.^16^ Para a comparação com as 25 amostras de leite aditivado, foram dosados os macronutrientes de 20 amostras de leite humano sem aditivo. Os teores de minerais foram comparados com os teores descritos por Palhares et al., em 1987.^17^ O valor energético das amostras foi calculado em calorias com a multiplicação de cada grama de carboidrato e de proteína por quatro e cada grama de gordura por nove.

Durante a produção do aditivo, houve a preocupação de manter a qualidade físico-química e o menor grau de contaminação microbiológica, uma vez que essa pode alterar os aspectos físico-químicos do leite. Para isso, foram tomados os seguintes cuidados: os recipientes que armazenavam o leite durante as duas fases de preparo, bem como a pipeta volumétrica, eram autoclavados; o fracionamento do leite para preparo e diluição do suplemento foi feito após desinfecção das mãos, com o uso de luvas, máscara, avental e gorro; as bordas dos frascos passavam pela chama do bico de Bunsen após serem abertos e antes de serem fechados; o banho-maria usado no descongelamento das amostras foi higienizado previamente e a água trocada a cada preparo; a cápsula de vácuo do liofilizador foi higienizada antes e após ser usada; o aditivo liofilizado ficou armazenado tampado, sob congelamento, no mesmo recipiente em que foi liofilizado até ser diluído. Neste trabalho, os RNMBP não foram alimentados com o leite aditivado e, por isso, o leite aditivado não passou por análise microbiológica. Porém, o aditivo foi elaborado para ser adicionado ao leite humano cru que deve ser pasteurizado após ser aditivado e passar pelas análises microbiológicas de rotina do banco de leite humano antes de ser oferecido aos recém-nascidos.

Em trabalhos experimentais os grupos comparados devem ter um tamanho amostral que seja suficiente para identificar a diferença entre eles, ou seja, o tamanho amostral se relaciona com a variação esperada entre os grupos avaliados, além do intervalo de confiança.^18^ Assim, o tamanho amostral neste trabalho foi definido com base em pesquisas da área e na diferença esperada entre os grupos analisados. Para comparar os teores nutricionais do leite humano aditivado com o leite humano sem aditivos foi usado o teste não paramétrico de Wilcoxon no GraphPad Software 2013 (GraphPad Software, Inc^(c)^, CA, EUA). O nível de significância estabelecido foi de 5%.

## Resultados

A média, o desvio padrão, o valor mínimo e o máximo de lactose, proteínas, lipídios, energia, osmolalidade, sódio, potássio, cálcio e fósforo do leite humano com aditivo homólogo estão referidos na tabela 1. Na tabela 2 estão apresentadas as médias dos teores nutricionais no leite humano com aditivo homólogo em comparação com o leite humano sem aditivo.

**Tabela 1:** Teores de lactose, proteínas, lipídios, energia, osmolalidade, sódio, potássio, cálcio e fósforo do leite humano com aditivo homólogo

Nutriente	Média	Desvio padrão	Mínimo	Máximo
Lactose (g/dL)	9,22	1,00	7,80	11,02
Proteína (g/dL)	2,20	0,36	1,60	2,90
Lipídios (g/dL)	2,91	0,57	1,81	4,01
Energia (kcal/dL)	71,93	8,69	54,88	89,45
Osmolalidade (mOsm/KgH_2_O)	389,60	32,40	335,00	438,00
Sódio (mEq/dL)	2,04	0,45	1,30	2,60
Potássio (mEq/dL)	1,42	0,15	1,22	1,66
Cálcio (mEq/dL)	43,44	2,98	38,07	48,21
Fósforo (mEq/dL)	23,69	1,24	21,72	25,51

**Tabela 2:** Teores nutricionais médios de leite humano com aditivo homólogo (LH + AH) e de leite humano de banco de leite (LH)

Nutriente	LH + AH	LH	Aumento n° de vezes	*p*
Lactose (g/dL)	9,22	6,78	1,36	<0,001
Proteína (g/dL)	2,20	1,13	1,95	<0,001
Lipídios (g/dL)	2,91	1,49	1,95	<0,001
Energia (g/dL)	71,93	45,06	1,6	<0,001
Osmolalidade (mOsm/KgH_2_O)	389,6	267,70	1,45	<0,001
Sódio (mEq/dL)	2,04	0,93^17^	2,19	<0,001
Potássio (mEq/dL)	1,42	1,18^17^	1,2	<0,011
Cálcio (mEq/dL)	43,44	24,71^17^	1,76	<0,001
Fósforo (mEq/dL)	23,69	10,75^17^	2,2	<0,001

## Discussão

A suplementação do leite humano com aditivos surgiu com o objetivo de adequá-lo às necessidades nutricionais do recém-nascido pré-termo (RNPT) de muito e extremo baixo peso. O uso de fortificantes comerciais e técnicas de lactoengenharia do leite humano, entre elas a estruturação adequada dos bancos de leite humano, a quebra de glóbulos de gordura do leite humano por ultrassom e a concentração do leite humano por meio da evaporação dos leites estocados nesses bancos renovaram o entusiasmo com o uso do leite humano para essas crianças.^19^

A técnica descrita neste estudo foi planejada para minimizar as etapas de manipulação, mudanças de temperatura e do estado físico do leite para melhor conservação dos nutrientes. A manipulação do leite é feita somente nas etapas de acondicionamento nos tubos plásticos cônicos e retirada do sobrenadante. Quanto às mudanças de temperatura, antes do processo de produção do aditivo, ele é congelado e descongelado somente uma vez durante o processo (na retirada do sobrenadante, após precipitação da lactose), quando é novamente congelado, liofilizado à temperatura de -40 °C e mantido sob congelamento até ser reconstituído em leite humano. Diferente das técnicas descritas em trabalhos semelhantes.^11^^,^ 12 ^,^ 13 ^and^ 14

A concentração de nutrientes no leite humano e o uso de aditivos levam ao aumento da osmolalidade. Por isso, parte da lactose foi retirada no processo de precipitação. O teor de lactose, por ser um dissacarídeo, aumenta a osmolalidade da solução.^20^ ^and^ 21 Por outro lado, a lactose aumenta a absorção de cálcio e favorece a fermentação, que diminui a obstipação intestinal.^22^ ^and^ 23 O teor médio de lactose no leite humano aditivado foi de 9,2 g/dl, de acordo com a recomendação, que é de 3 a 12 g/dL. Com o início da alimentação enteral mínima, a atividade da lactase aumenta rapidamente. Apesar do aumento no teor de lactose, a osmolalidade encontrada foi de 389,6mOsm/KgH_2_O. A osmolalidade do alimento destinado ao prematuro deve ser inferior a 450mOsm/kgH_2_O.^24^ Santos,^12^ em 1997, alimentou RNMBP com leite humano adicionado de aditivo homólogo com teor de lactose semelhante a esse e relatou boa tolerância gastrointestinal. Assim, o teor do encontrado neste trabalho parece ser adequado para alimentar RNMBP.

Os valores de proteínas encontrados no leite humano aditivado atendem à recomendação da AAP,^25^ em 1985, de 2,9-3,3 g/100 kcal, e da Sociedade Canadense de Pediatria,^26^ que recomendou 2,7-3,5 g/kg/dia em 1995. Com uma oferta de leite aditivado de 150-200 mL/kg ao dia, atinge-se ingestão de 3,3-4,4 g/kg de proteína ao dia, coincidente com o intervalo de 3,5-4,0 g/kg/dia recomendado pelo Comitê da Sociedade Europeia de Gastroenterologia Hepatologia e Nutrição Pediatrica (ESPGHAN) em 2010.^27^

O teor de sódio encontrado no leite aditivado também atende à recomendação da AAP para fórmulas infantis, que é de 2,5-3,5mEq/kg/dia, quando oferecidas 120 kcal/kg/dia,^25^ e da ESPGHAN^27^ (2,7-4,6mEq/100 kcal). Se a capacidade gástrica da criança permitir, a ingestão de um volume de leite que oferte 4mEq/kg/dia de sódio é recomendada para prevenção de hiponatremia.^25^ Já quanto ao potássio, o leite humano aditivado neste estudo ofereceu 1,42mEq/dL de potássio. O requerimento de potássio estimado pode ser suprido com o aleitamento materno que contém 1,25-1,60mEq/dL.^28^

A presença de cálcio no leite humano difere das fórmulas infantis, não somente em quantidade, como também nas espécies químicas constituintes, devido às diferenças na fração proteica do leite humano e de vaca. Nas fórmulas, o cálcio está associado principalmente à caseína. No leite humano, uma proporção elevada de cálcio forma parte da fração lipídica. Na fração aquosa, a maior parte do cálcio está associada às proteínas do soro e a compostos de baixo peso molecular. No leite humano há pouco cálcio ligado à caseína. Essas diferenças na estrutura química das espécies constituintes do conteúdo de cálcio dos leites explicam a elevada biodisponibilidade de cálcio do leite humano.^28^ Apesar do aumento significativo nos teores de cálcio e fósforo do leite humano aditivado em relação leite humano sem aditivos, esses teores não atendem às recomendações para recém-nascidos de muito baixo peso que, segundo a ESPGHAN, são de 20-140 mg/kg/dia de cálcio e 60-90 mg/kg/dia de fósforo^27^ e, segundo a AAP, são de 200 a 250 mg/kg/dia de cálcio e de 110 a 125 mg/kg/dia de fósforo.^25^ Ainda assim, esse aumento significativo, aproximadamente o dobro do leite humano sem aditivos, é vantajoso pela maior biodisponibilidade do cálcio e do fósforo do leite humano, comparado aos suplementos comerciais. Além disso, a aquisição de suplementos de cálcio e fósforo isolados é mais barata do que a aquisição de suplementos com multinutrientes.

Devido à incorporação de gordura no feto ocorrer no último trimestre gestacional, o RNMBP é vulnerável à falta de lipídios. A gordura da dieta do RNMBP deve atender às necessidades de ácidos graxos e prevenir a falta de calorias.^29^ A quantidade de gordura do leite humano com aditivo homólogo em g/100Kcal foi de 4 g, próximo das recomendações da AAP, 4,5-6 g/100kcal,^25^ e da ESPGHAN,^27^ 4,4-6 g/100 kcal. Os teores de gordura do leite humano com aditivo homólogo e do leite humano sem aditivos apresentados nos resultados são mais baixos do que a média de gordura do leite humano descrita na literatura. Isso ocorreu porque os leites usados não foram selecionados segundo seus valores calóricos, devido à dificuldade de ter leite excedente no banco de leite humano para a pesquisa. Apesar disso, o aditivo feito com esses leites "magros" foi capaz de melhorar significativamente o conteúdo nutricional do leite humano de banco para os RNMBP.

O leite produzido pela mãe do recém-nascido pré-termo apresenta composição diferenciada do leite maduro no aporte proteico energético e de constituintes imunológicos nas primeiras semanas de produção. Essas modificações tornam o leite materno adaptado às necessidades do prematuro. Nas primeiras quatro semanas, o leite humano pré-termo contém maior concentração de nitrogênio, proteína com função imunológica, lipídios totais, ácidos graxos de cadeia média, vitaminas, cálcio, sódio e energia do que os de mães de recém-nascidos a termo. Quanto maior o grau de prematuridade, maior é o teor proteico e de lipídios.^1^ ^and^ 30 Com exceção do cálcio e do fósforo, os teores nutricionais avaliados no leite aditivado atendem às recomendações para a nutrição de RNMBP e sua composição é semelhante à do leite humano pré-termo, indicado para alimentação dessas crianças. O resultado obtido no presente estudo é similar ao do leite humano aditivado com os aditivos produzidos por Thomaz et al.^14^ e tem maior teor de proteínas do que os produzidos por Santos^12^ e Valentini.^13^

As principais limitações na metodologia deste estudo são a sua reprodubilidade, pois os valores de composição nutricional variam de acordo com o leite humano usado; o uso de diferentes *pools* de leite humano na produção do aditivo, na diluição e na comparação de suas composições nutricionais; e a comparação do teor de minerais com dados de outro estudo. Tais limitações estão justificadas abaixo.

Ao reproduzir a metodologia exposta neste trabalho, os resultados obtidos da composição nutricional do leite humano com ou sem aditivo podem variar por causa da composição nutricional do leite humano usado, que sofre variações ao longo do dia, de uma mama para outra, do início para o fim da mamada e durante o tempo de lactação. Porém, o principal objetivo deste estudo é mostrar que a técnica para produção do aditivo é capaz de tornar o leite de banco de leite humano mais adequado à alimentação dos RNMBP sem impor critérios de seleção e exclusão de leite, além daqueles que garantem a segurança sanitária. Isso porque nos bancos de leite humano não há normalmente leite humano excedente. Assim, uma técnica que imponha critérios de seleção do leite disponível para a produção do aditivo não seria viável. Devido a essa variação de disponibilidade de leite no banco de leite humano, neste estudo o leite usado na produção do aditivo, na diluição e na comparação de suas composições nutricionais não se originou do mesmo *pool*, mas representou valores médios de diferentes amostras.

A comparação do teor de minerais do leite humano com aditivo homólogo com leite humano sem aditivos foi feita com os dados publicados em outro trabalho, porque a composição nutricional do leite humano sem aditivos é bem descrita nas publicações.

Quanto à viabilidade da produção do aditivo, o custo único e inicial para equipar o banco de leite humano e produzir o aditivo homólogo é de U$ 19 mil para aquisição da centrífuga refrigerada e do liofilizador de bancada. Considerando que o preço de mercado da caixa do aditivo comercial com 70 flaconetes é de R$ 115 e que um flaconete aditiva 20 mL de leite humano, o custo para alimentar uma criança de 1.000 g com um volume diário de 150 mL/kg por 30 dias será de R$ 370,00. Para alimentar 10 crianças por dia com esse volume durante um ano serão gastos R$ 44.400. Sendo assim, uma unidade de terapia neonatal que atende 10 crianças por mês, se usar o volume proposto, terá gastado em um ano o mesmo valor necessário para equipar o banco de leite humano para produzir o aditivo homólogo.

O aditivo homólogo tem vantagens sobre aditivos comerciais por oferecer proteínas de alto valor biológico e nutrientes do próprio leite humano. A técnica pode ser implantada nos bancos de leite humano e apresenta viabilidade financeira para países em desenvolvimento por requerer apenas investimento inicial para aquisição dos equipamentos e por diminuir os gastos com aditivos comerciais.
